# Phytochemical Analysis and Biological Activities of *Cola nitida* Bark

**DOI:** 10.1155/2015/493879

**Published:** 2015-02-12

**Authors:** Durand Dah-Nouvlessounon, Hubert Adoukonou-Sagbadja, Nafan Diarrassouba, Haziz Sina, Adolphe Adjanohoun, Mariam Inoussa, Donald Akakpo, Joachim D. Gbenou, Simeon O. Kotchoni, Mamoudou H. Dicko, Lamine Baba-Moussa

**Affiliations:** ^1^Laboratoire de Biologie et de Typage Moléculaire en Microbiologie, Faculté des Sciences et Techniques, Université d'Abomey-Calavi, 05 BP 1604 Cotonou, Benin; ^2^Laboratoire des Ressources Génétiques et d'Amélioration des Espèces, Département de Génétique et des Biotechnologies, FAST, Université d'Abomey-Calavi, BP 526 Cotonou, Benin; ^3^Université Péléforo Gon Coulibaly de Korhogo UFR des Sciences Biologiques, BP 1328 Korhogo, Cote d'Ivoire; ^4^Centre de Recherches Agricoles Sud, Institut National des Recherches Agricoles du Bénin, Attogon, BP 884 Cotonou, Benin; ^5^Laboratoire de Pharmacognosie et des Huiles Essentielles, FSS/UAC, 01 BP 4521 Cotonou, Benin; ^6^Department of Biology and Center for Computational and Integrative Biology, Rutgers University, 315 Penn Street, Camden, NJ 08102, USA; ^7^Laboratoire de Biochimie Alimentaire, Enzymologie, Biotechnologie Industrielle et Bioinformatique, UFR/SVT Université de Ouagadougou, 03 BP 7021 Ouagadougou 03, Burkina Faso

## Abstract

Kola nut is chewed in many West African cultures and is used ceremonially. The aim of this study is to investigate some biological effects of *Cola nitida*'s bark after phytochemical screening. The bark was collected, dried, and then powdered for the phytochemical screening and extractions. Ethanol and ethyl acetate extracts of *C. nitida* were used in this study. The antibacterial activity was tested on ten reference strains and 28 meat isolated *Staphylococcus* strains by disc diffusion method. The antifungal activity of three fungal strains was determined on the Potato-Dextrose Agar medium mixed with the appropriate extract. The antioxidant activity was determined by DPPH and ABTS methods. Our data revealed the presence of various potent phytochemicals. For the reference and meat isolated strains, the inhibitory diameter zone was from 17.5 ± 0.7 mm (*C. albicans*) to 9.5 ± 0.7 mm (*P. vulgaris*). The MIC ranged from 0.312 mg/mL to 5.000 mg/mL and the MBC from 0.625 mg/mL to >20 mg/mL. The highest antifungal activity was observed with *F. verticillioides* and the lowest one with *P. citrinum*. The two extracts have an excellent reducing free radical activity. The killing effect of *A. salina* larvae was perceptible at 1.04 mg/mL. The purified extracts of *Cola nitida*'s bark can be used to hold meat products and also like phytomedicine.

## 1. Introduction

The African continent has a huge biodiversity with a high number of plants used for medicinal [1], food and in traditional ceremonies. In recent years, there has been a gradual revival of interest in the use of medicinal plants in developing nations [2]. Despite the existence of hospitals in developing countries and laws that consider traditional medicine as an illegal act, the valorization of the traditional medicine has become an early criterion of identity as well as rights of health and education [3]. In the same way, it was reported that about 80% of Africans use medicinal plants to treat various diseases [4]. Among the medicinal plants, cola nut (*Cola nitida*) highly spreads in Africa and particularly in Benin.

Indeed,* C. nitida* is a plant native to tropical West Africa and belongs to Sterculiaceae family [5]. This plant is nowadays cultivated from Senegal to Nigeria, in the West Indies and South America [6]. In West African's forest areas, cola is perhaps second in importance to the palm tree as an indigenous cash crop [7]. Cola nut has been an important article of international trade in many parts of Africa [8]. The nuts of* C. nitida* contain about two percent of caffeine and are chewed by many people as a stimulant. It is a very special and important item used in social and ceremonial activities by Africans. The nuts of cola also have industrial usage for the production of drugs, soft drinks, wines, candies, and beverages [9] such as Coca-Cola and Pepsi-Cola [10]. It has many pharmacological properties and contains some active principles: it prevents sleep, thirst, and hunger and acts as an antidepressant [7]. The cola nuts are source of antioxidants and contain a wide array of complex secondary plant metabolites such as theobromine, d-catechin, L-epicatechin, and kolatin [11]. The use of the plant in the treatment of certain diseases has been reported by several authors [12, 13].

Nowadays there exist more than 250 types of infections caused by bacteria and fungi [14, 15]. Among these microorganisms, we find the* Pseudomonas*,* Escherichia coli* [16], and bacteria of the genus* Staphylococcus* that are known to be one of the main elements of human physiological flora [17] and are responsible of many diseases [18–20]. In the rank of fungi, the moulds producing toxins are mainly of genus* Aspergillus*,* Penicillium*, and* Fusarium* [21]. With the advent of modern medicine and for the treatment of infections, abusive and often uncontrolled use of antibiotics brings up a phenomenon of resistance in most of the bacteria and fungi. Beyond infectious diseases, oxidative stress is a very serious phenomenon that can trigger molecular and cellular events in the body. The consequences are multiple, such as cancer [22], cerebral and cardiovascular diseases, diabetes, and hypertension [23].

To face these health problems (resistance of microorganisms and natural phenomenon of production in the body of the free radicals), the track of medicinal plants deserves to be explored. In this direction in Benin, several ethnobotanical studies [24–26] have focused for several years on identifying medicinal species. Others have demonstrated the efficacy of medicinal plants in the fight against certain fungal strains [27], and pathogenic Gram-positive and Gram-negative bacteria involve several pathologies [28]. Furthermore, in Benin, very little study has been conducted on* C. nitida*, and the references which relate to its phytochemical composition and its biological properties are quasi-nonexistent. In the same way the work realized on the species in other countries concerns seed; those concerning species bark are rare and the results are disparate. The aim of this study was to make the phytochemical screening of* C. nitida* bark and to investigate* in vitro* some biological (antimicrobial, antifungal, and cytotoxic) activities of its extracts.

## 2. Material and Methods

### 2.1. Collection of Plant Material

The bark of* C. nitida* was collected in the village of Aglogbè (commune of Adjarra: 6°24′0′′N, 2°12′0′′E), Department of Oueme, southern Benin. The plants materials were air-dried at 25°C–30°C for two weeks, ground, and sieved into a bark powder. The smooth powder was stored in airtight glassware and kept in darkness at −20°C until use.

### 2.2. Phytochemical Profiling

The phytochemical profiling of the bark of* C. nitida* to determine the major constituents (nitrogenous, polyphenolic, and terpenic compound and glycosides) was done according to Houghton and Raman [29].

### 2.3. Preparation of Ethanol and Ethyl Acetate Extracts

These extracts were made using an adapted method of the one described by Sanogo et al. [30] and N'Guessan et al. [31]. This method consisted of macerating 50 g of* C. nitida* powders in 500 mL of 96% ethanol for 72 hours. The obtained extract was filtered thrice using Whatman filter paper. Half of the filtrate was directly dried at 40°C to obtain the ethanolic extract of* C. nitida*. To the second half of the filtrate, 200 mL of H_2_O and 100 mL of ethyl acetate were added. The solution was gently mixed and left settled until we obtain two phases (about 45 min). The lower phase was collected and dried as described previously to obtain the ethyl acetate extract. The alcoholic and ethyl acetate extracts were stored in labeled sterile bottles and kept at −20°C until further use.

### 2.4. Microorganism's Cultures

The tested microorganisms include ten references, twenty height* Staphylococcus* meat isolated strains, and three fungal strains (*Penicillium citrinum*,* Aspergillus tamarii*, and* Fusarium verticillioides*). The three fugal strains were part of the microorganisms isolated in the Beninese traditional cheese wagashi by Sessou et al. [27]. The reference strains were* Escherichia coli* ATCC 25922,* Staphylococcus aureus* ATCC 29213,* Staphylococcus epidermidis* T22695,* Pseudomonas aeruginosa* ATCC 27853,* Proteus mirabilis* A24974,* Micrococcus luteus* ATCC 10240,* Proteus vulgaris* A25015,* Streptococcus oralis*,* Enterococcus faecalis* ATCC 29212, and* Candida albicans* MHMR. The* Staphylococcus* strains used in this study were those isolated from three different meat products in Ivory Coast by Attien et al. [32] and stored in the Laboratory of Biology and Molecular Typing in Microbiology (University of Abomey-Calavi, Benin).

### 2.5. Antimicrobial Activity

#### 2.5.1. Sensitivity Test

The disc diffusion method [33] was used to screen the antimicrobial activity. Briefly, two to three sterile paper discs (6 mm as diameter) were lodged, under aseptic conditions, on Mueller Hinton agar Petri dish previously flooded with the appropriate bacterial culture (adjusted to 0.5 McFarland standard). The discs were aseptically impregnated with 25 *μ*L of* C. nitida* extract solution (20 mg/mL). These dishes were kept for 15–30 min at room temperature before incubation at 37°C for 24 and 48 hours.

After the incubation period, the dishes were examined for inhibitory zones [34]. Each sample was used in triplicate for the determination of antibacterial and antifungal activity. Blank disc impregnated with solvent was used as negative control.

#### 2.5.2. Determination of Minimum Inhibitory Concentrations (MIC)

The minimum inhibitory concentrations (MIC) of crude extract of plants were performed by macrodilution method [35]. First, the extracts were diluted in sterilized distilled water to the highest concentration of 20 000 *μ*g/mL and then nine dilutions were performed to obtain the concentrations of 10 000 *μ*g/mL, 5 000 *μ*g/mL, 2 500 *μ*g/mL, 1 250 *μ*g/mL, 625 *μ*g/mL, 312.5 *μ*g/mL, 156.25 *μ*g/mL, 78.12 *μ*g/mL, and 39.06 *μ*g/mL in screw tube. To 1 mL of the above concentrations was added 1 mL of the bacteria inoculum (10^6^ UFC/ml) to obtain 2 mL as a final volume. Culture medium without samples and others without microorganisms were used in the tests as control. Tubes were incubated at 37°C for 18–24 hours and growth was indicated by turbidity. The MIC is the lowest concentration of the compound at which the microorganism tested does not demonstrate visible growth (turbidity).

#### 2.5.3. Minimum Bactericidal Concentration (MBC)

The minimum bactericidal concentration (MBC) of the tested microorganisms was determined by subculturing the test dilutions onto a fresh solid medium and further incubation for 18–24 h. The highest dilution that yielded no bacterial growth on solid medium was taken as MBC [36].

### 2.6. Evaluation of the Cytotoxicity Activity of* Cola nitida*'s Bark Extracts

The cytotoxic effect of the extracts was evaluated according to an adaptation of the method described by Kawsar et al. [37]. The tests are carried out twice on 72 h larvae of* Artemia salina*. Briefly, a test was constituted of 16* A. salina* larvae in a 2 mL solution containing 1 mL of the extract tested concentration and 1 mL of sea water. The number of surviving larvae is counted after incubation (24 h) and the DL_50_ was calculated using the regression line obtained from the surviving larvae in function of the extracts concentration representation.

### 2.7. Antifungal Activity

The* in vitro* antifungal activity of the extracts was evaluated according to the method previously described by Kumar et al. [38] and Dohou et al. [39]. The assay was performed on the Potato-Dextrose Agar medium. Briefly, the extracts (20 mg/mL) used for the antifungal activity were dissolved with sterilized distillated water or if necessary with a water-ethanol mixture (60 : 40). One mL of the dissolved extract (20 mg/mL) was thoroughly mixed with 10 mL of the sterilized Potato-Dextrose Agar medium before it was transferred to sterile Petri dishes for solidification. After the medium solidification, a sterile 6 mm disc treated with fungal strain was placed in each Petri plate. Each treatment was replicated twice. Plates were incubated at 25 ± 1°C for 5 days. Fungal radial growth was measured by averaging the two diameters taken from each colony. Percentage growth inhibition of the fungal colonies was calculated using the formula

(1) Inhibition  Percentage (%) =Control's  growth−Treatment's  growth  Control's  growth×100.

### 2.8. Antioxidant Activity Determinations

The antioxidant activity was measured using both DPPH (2,2-diphenyl-1-picrylhydrazyl) and ABTS [2,2′-azinobis(3-ethylbenzothiazoline-6-sulfonic acid)] methods.

The ABTS assay was conducted according to the method described by Re et al. [40]. The working solution of ABTS^+^ (10 mg of ABTS, 2.6 mL of deionized water, and 1.72 mg of potassium persulphate) was left to stand at room temperature for 12 h in the dark before use. This solution was diluted with ethanol until obtaining an absorbance of 0.70 ± 0.02 at 734 nm. Twenty *μ*L of each extract sample (1 mg/mL) was diluted with a fresh prepared ABTS solution to a total volume of 1 mL. All the assays were performed in triplicate, the absorbance was read after 15 min in dark at 734 nm, and the reference molecule was ascorbic acid. The concentration of compounds with a capability to reduce ABTS^+^ radical cation is expressed as *μ*mol equivalent ascorbic acid (*μ*mol EqAA) per gram of dry extract using the following formula used by Guenne et al. [41].

The DPPH method was conducted by adaptation as described by Scherer and Godoy [42]. Equal volumes (100 *μ*L) of DPPH (50 *μ*M) and plant extracts (200 *μ*g·mL^−1^) were mixed in a 96-well microplate and allowed to stand in darkness for 20–30 min at room temperature. Then, the absorbance was read at 517 nm and the blank was a mixture of methanol and DPPH (v : v). The inhibitory percentage of DPPH radical indicating the antioxidant activity of extracts and quercetol, gallic acid was obtained using the formula established by Schmeda-Hirschmann et al. [43].

The concentration providing 50% inhibition (IC_50_) was determined graphically using a calibration curve in the linear range by plotting the extract concentration and the corresponding scavenging effect. Antioxidant activity index (AAI) was calculated according to the formula used by Scherer and Godoy [42].

### 2.9. Statistical Analysis

All experiment was done in triplicate and data thus obtained were reported as a mean ± standard deviation (SD). The data were analyzed using GraphPad Prism 5 software. Differences of *P* < 0.05 were considered significant.

## 3. Results

### 3.1. Phytochemical Screening

The result of phytochemical screening of* C. nitida*'s bark powder revealed the presence of various potent phytochemicals such as tannins, saponins, and flavonoids (Table 1).

### 3.2. Antibacterial Activity

The results of antibacterial activity using ethanol and ethyl acetate extract of* C. nitida* (20 mg/mL) showed a various effect on reference and meat isolated strains. Indeed, among the reference strains, we observed that there was an antimicrobial activity on all the strains except* E. coli* (Table 2). Then, 90% (9/10) of the tested reference strains were sensitive to* C. nitida*'s ethanol and ethyl acetate extracts.

**Figure 1:**
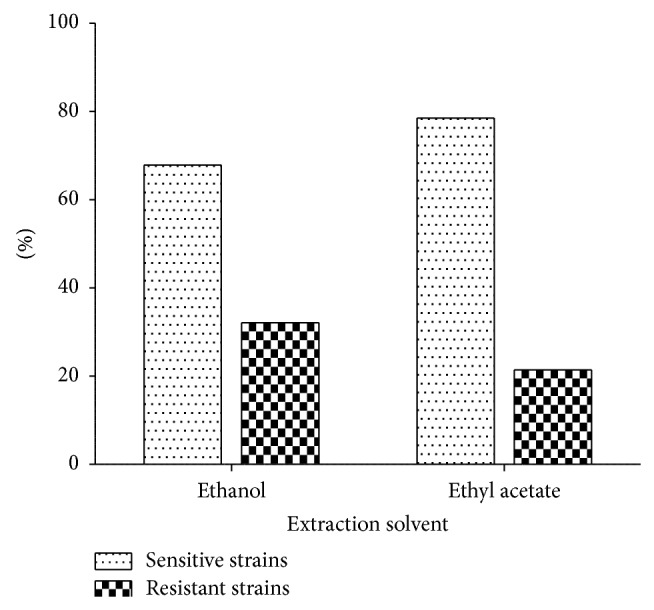
Antimicrobial effect of* C. nitida* extracts on* Staphylococcus* strains isolated from meat.

Concerning the meat isolated strains, our data displays that 78.57% (22/28) of the tested strains were sensitive to ethyl acetate extracts against 67.85% (19/28) for the ethanol extract (Figure 1).

#### 3.2.1. Susceptibility

**Figure 2:**
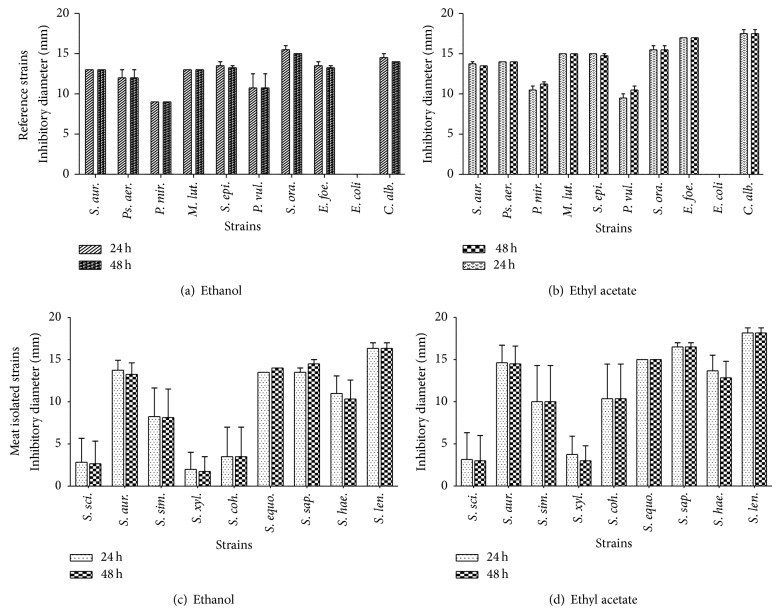
Medium inhibitory diameter zone of* C. nitida* extracts on reference and meat isolated* Staphylococcus* strains after 24 h and 48 h of incubation. Reference strains are the following:* S. aur.*:* Staphylococcus aureus*,* M. lut.*:* Micrococcus luteus*,* S. epi.*:* Staphylococcus epidermidis*,* S. ora.*:* Streptococcus oralis*,* Ps. aer.*:* Pseudomonas aeruginosa*,* E. foe.*:* Enterococcus faecalis*,* P. vul.*:* Proteus vulgaris*,* E. coli*:* Escherichia coli*,* C. alb.*:* Candida albicans*,* P. mir.*:* Proteus mirabilis*; meat isolated strains are the following:* S. sci.*:* S. sciuri*,* S. aur.*:* S. aureus*,* S. sim.*:* S. simulans, S. xyl.*:* S. xylosus, S. coh.*:* S. cohnii*,* S. equ.*:* S. equorum*,* S. sap.*:* S. saprophyticus*,* S. hae.*:* S. haemolyticus*, and* S. len.*:* S. lentus*.

The inhibitory diameter zones of the sensitive strains vary according to species and the kind of extract. Thus, for the reference strains, Figure 2 indicated that there was not a significant variation of the diameter of inhibitory zones according to the time (*P* > 0.05) with both ethanol extract (Figure 2(a)) and ethyl acetate extract (Figure 2(b)). There was not also any significant difference comparing the inhibition diameters of the meat isolated* Staphylococcus* strains (Figures 2(c) and 2(d)).

**Figure 3:**
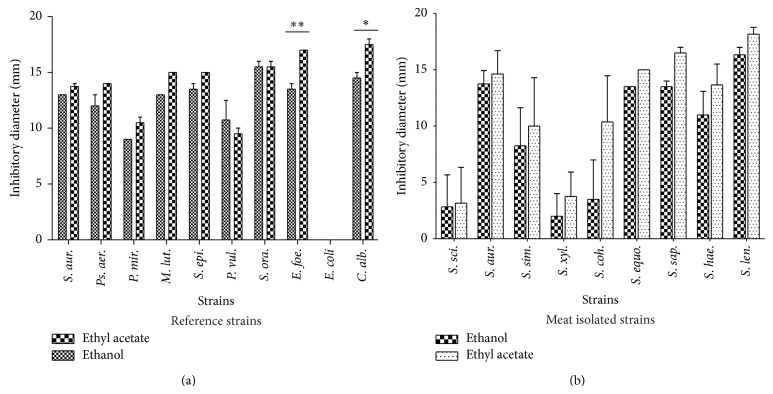
Comparison of medium inhibitory diameter of* C. nitida*'s ethyl acetate extract and ethanol extract on reference strains and meat isolated* Staphylococcus* strains. Reference strains are the following:* S. aur.*:* Staphylococcus aureus*,* M. lut.*:* Micrococcus luteus*,* S. epi.*:* Staphylococcus epidermidis*,* S. ora.*:* Streptococcus oralis*,* Ps. aer.*:* Pseudomonas aeruginosa*,* P. vul.*:* Proteus vulgaris*,* E. coli*:* Escherichia coli*,* C. alb.*:* Candida albicans*,* P. mir.*:* Proteus mirabilis*; meat isolated strains are the following:* S. sci.*:* S. sciuri*,* S. aur.*:* S. aureus*,* S. sim.*:* S. simulans*,* S. xyl.*:* S. xylosus*,* S. coh.*:* S. cohnii*,* S. equ.*:* S. equorum*,* S. sap.*:* S. saprophyticus*,* S. hae.*:* S. haemolyticus*, and* S. len.*:* S. lentus.* ^*^ *P* < 0.05; ^**^ *P* < 0.01.

Figure 3 shows that, in most of the cases, the susceptibility of the tested reference strains varied depending on the kind of solvent used for the extraction but their effect was not statistically different (*P* > 0.05). Globally, in both reference strains (Figure 3(a)) and meat isolated ones (Figure 3(b)), the ethyl acetate extracts were more efficient. Thus, in the reference strains we observed the highest diameter on* C. albicans* (17.5 ± 0.7 mm) and the lowest on* P. vulgaris* (9.5 ± 0.7 mm). But, only with* S. oralis*, the same diameters were obtained with both ethanol and ethyl acetate extracts (15.5 ± 0.7 mm). With the meat isolated* Staphylococcus *strains, the highest diameter was observed on* S. lentus* using both ethyl acetate extract (18.19 ± 1.04 mm) and ethanol extract (16.33 ± 1.15 mm).

#### 3.2.2. Determination of Minimum Inhibitory Concentrations (MIC)

Table 3 shows the minimum inhibitory concentrations (MIC) of* C. nitida*'s bark extracts on ten reference strains and on twenty height strains of nine* Staphylococcus* species isolated from meat products.

Considering the reference strains, the mean concentration values widely vary, depending on the tested strains, and ranged from 0.312 mg/mL to 5.000 mg/mL. For ethyl acetate extract, the lowest MIC was 0.312 mg/mL with* Candida albicans*. Five strains (*Staphylococcus aureus*,* Pseudomonas aeruginosa*,* Proteus mirabilis*,* Proteus vulgaris*, and* Enterococcus faecalis*) display MIC of 1.25 mg/mL whereas the highest concentration (2.5 mg/mL) value was recorded with* Micrococcus luteus*. Considering the ethanol extract, the largest MIC was observed on* Micrococcus luteus* (5 mg/mL), while the most sensitive strains displaying the lowest MIC (0.312 mg/mL) were* Staphylococcus aureus*,* Streptococcus oralis*, and* Enterococcus faecalis*. Three other strains (*Proteus mirabilis*,* Staphylococcus epidermidis*, and* Proteus vulgaris*) had 1.25 mg/mL as MIC value.

For the meat isolated strains, it globally appears that the mean values ranged from 0.078 mg/mL to 1.250 mg/mL. For ethyl acetate extract, the lowest MIC was 0.078 mg/mL with three species of* Staphylococcus* (*S. equorum*,* S. saprophyticus*, and* S. lentus*) and the largest MIC (0.625 mg/mL) was observed with* S. haemolyticus*. Considering the ethanol extract, the largest MIC (1.25 mg/mL) was observed on* S. aureus* and* S. xylosus* while the most sensitive strain displaying the lowest MIC (0.078 mg/mL) was* S. haemolyticus*. The other values for the MIC were 0.156 mg/mL (*S. equorum* and* S. lentus*), 0.312 mg/mL (*S. simulans* and* S. saprophyticus*), and 0.625 mg/mL (*S. sciuri* and* S. cohnii*).

#### 3.2.3. Determination of the Minimum Bactericidal Concentration (MBC)

Table 4 presents the MBC of* Cola nitida*'s bark extract on ten reference strains and on twenty height meat isolated* Staphylococcus* strains.

For the reference strains, results show that the MBCs varied (from 1.25 mg/mL to** >**20 mg/mL) according to the bacterial strains and the kind of extract. With the ethanol extract, the lowest MBC was 2.5 mg/mL (*S. aureus*,* P. aeruginosa*,* S. epidermidis*,* E. faecalis*, and* C. albicans*) whereas the highest was >20 mg/mL (*M. luteus*). With the ethyl acetate extract, the highest MBC was >20 mg/mL (*M. luteus*) and the lowest (1.25 mg/mL) was recorded on* S. aureus* and* E. faecalis*.

Considering the meat isolated strains, our data displays that the MBC of the tested extracts varied from 0.625 mg/mL to 5 mg/mL depending on the tested* Staphylococcus* species. Using ethanol extract, the lowest MBC (0.625 mg/mL) was observed on* S. equorum* while the highest MBC (5 mg/mL) was obtained with this extract on* S. cohnii* and* S. xylosus*. With the ethyl acetate extract, the largest MBC (5 mg/mL) was obtained on* S. xylosus* while the lowest MBC (0.625 mg/mL) was obtained on* S. sciuri*,* S. equorum*,* S. saprophyticus*, and* S. lentus*.

#### 3.2.4. Evaluation of Bactericidal and Bacteriostatic Effects of* Cola nitida* Bark Extracts

The ratio MBC/MIC was calculated to evaluate the kind of effect exerted by the* Cola nitida* bark extracts on the tested strains. Our data displays that the extracts have both bactericidal and bacteriostatic effects on reference and meat isolated strains. Thus, with the reference strains, the ethyl acetate extract has bactericidal effect on* S. aureus*,* P. aeruginosa*,* S. epidermidis*, and* E. faecalis*. The ethanol extract had a bactericidal effect on only* S. epidermidis* (Table 5). With the meat isolated* Staphylococcus strains*, we observed a bactericidal effect on* S. sciuri* (with ethyl acetate extract) and* S. aureus* (with ethanol extract), while all the other strains show the bacteriostatic effect in presence of the tested extracts (Table 5).

### 3.3. Antifungal Activity of* Cola nitida* Bark Extracts

**Figure 4:**
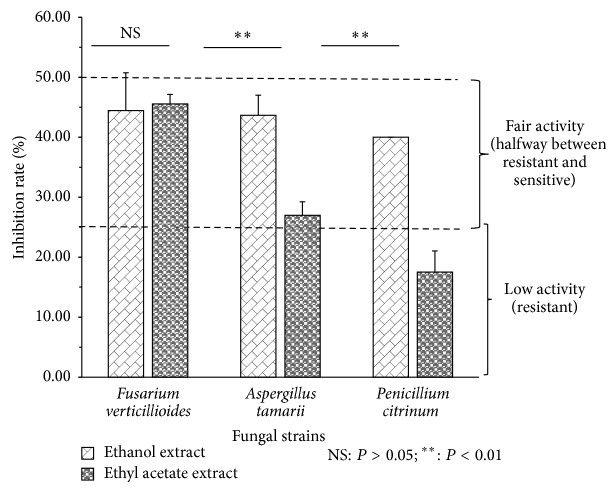
Inhibition rate of* Cola nitida*'s extracts in the fungal growth.

Figure 4 indicated that the antifungal activity using ethanol and ethyl acetate extracts of* C. nitida* (1.8 mg/mL) was statistically variable in regard of the used fungal strains (*P* = 0.0016). The inhibitory rate varies from 20 to 46.7%. Moreover, the antifungal effect varies according to the kind of extract (*P* = 0,007). Indeed, the interaction between the strains and the ethyl acetate extract displays a difference of action considering* F. verticillioides* and* A. tamari* (*P* < 0.01) and then* F. verticillioides* and* P. citrinum* (*P* < 0.001). With the ethanol extract, there was no statistical difference independently of the strains (*P* > 0.05).

### 3.4. Antioxidant Activity of* Cola nitida* Bark Extracts

Table 6 shows the radical scavenging activity by DPPH. Our data reveal that the IC_50_ of the ethanol extract (9.00 ± 1.73 *μ*g·*μ*L^−1^) was about two times higher than the one observed with the ethyl acetate extract (4.53 ± 0.98 *μ*g·*μ*L^−1^). Moreover, it appears that the IC_50_ values of the reference molecules (quercetin and gallic acid) are lower than the tested extracts. The ethyl acetate extract of* C. nitida* had an AAI value (11.02 ± 1.49 *μ*g·*μ*L^−1^) higher than the one observed with the ethanol extract (5.71 ± 1.23 *μ*g·*μ*L^−1^).

Table 6 shows also the antioxidant activity of* Cola nitida* bark extracts as the ability to reduce ABTS^∙+^ cation. This activity was determined from a linear regression curve (*y* = −0.001*x* + 0.5721, *R* ^2^ = 0.9715). Data in Table 6 indicate that the extracts of* Cola nitida* have more significant activity than the one obtained with ascorbic acid used as reference (35.02 ± 0.73 *μ*molEqAA·g^−1^). In addition our data indicate that the ethanol extract reduces more the ABTS^∙+^ cation than the DPPH one. Nevertheless, independently of the methods, the two* Cola nitida* extracts follow the same efficacy order.

### 3.5. Cytotoxicity Assay of* Cola nitida*'s Bark Extracts

**Figure 5:**
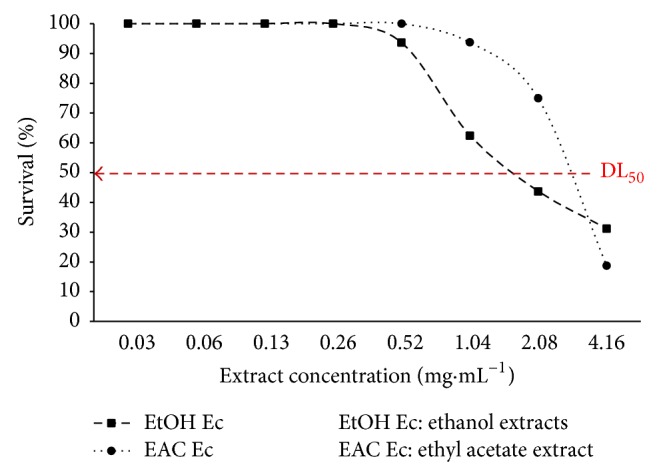
Variation of* A. salina* larval mortality according to* C. nitida* extracts concentration.

The bioassay to determine the lethality effect of* Cola nitida*'s bark extracts on* Artemia salina* was used to evaluate the cytotoxicity of our extracts. Thus, Figure 5 indicates the evolution of mortality according to the tested concentrations of our extracts. Indeed, for the ethanol extracts, the mortality of the* A. salina* larvae was observed from the concentration of 0.52 mg·mL^−1^ whereas the killing effect started to be perceptible at 1.04 mg·mL^−1^.

## 4. Discussion

The qualitative screening of* Cola nitida*'s bark extracts revealed the presence of various phytochemical components such as tannins, flavonoids, and saponins (Table 1). The presence of tannins in our tested extracts suggests the probable biological activities. Indeed, tannins are reported not only to promote tissue regeneration in case of superficial burn injury but also to have antibacterial, antiviral, antifungal, and antioxidant effects [44]. The presence of flavonoids in the extracts indicates their potentiality to reduce* in vitro* cholesterol agents and to induce an antifungal activity [44, 45]. Flavonoids are known to inhibit *α*-amylase activity which regulates the amount of glucose in the blood; therefore the extracts of* C. nitida* can be used as an antidiabetic. The presence of flavonoids and saponins has earlier been reported in 2009 by N'Guessan et al. [46] in Côte d'Ivoire during their work on the same plants. Nevertheless, we observed in our study the absence of triterpene and steroid and the presence of tannins whereas triterpene and steroid were observed without tannins in the same organ of the plant [46] and the other parts of the same plant [13, 47]. These observations may be probably due, in the case of the same organ, to the collection conditions such as origin of the plants, the conditions, and the periods of harvesting organs. We should notice that the environment may influence the synthesis and expression of phytochemical components in the plant [48–52]. Some plant physiologists went further saying that plants components can be produced only at a certain time and/or in a determined condition. For the same plant species, there is an unequal distribution of secondary metabolites through the organs.

Among the ten reference strains, at the unique concentration of 20 mg/mL, the ethanol and ethyl acetate extracts have inhibited the growth of yeast and Gram + and Gram − bacteria (Table 2). An antimicrobial effect was not observed on* E. coli* at the used concentration. This observation on the susceptibility of* E. coli* is different from those observed in 2011 by Indabawa and Arzai [53] during their study on the antibacterial activity on the seed of* C. nitida*. Indeed, those authors found that* C. nitida*'s seed aqueous extract inhibits the growth of* E. coli* at the concentration of 500 *μ*g/mL. That difference can be explained by the fact that we do not use the same organs.

Concerning the meat isolated* Staphylococcus strains*, our results indicate that the solvent plays a role in the extraction of active principles (Figure 1). The ethanol extracts are less effective than ethyl acetate extracts (*P* = 0.028) on meat isolated* Staphylococcus strains*. These results are similar to those of Bolou et al. [54] obtained during their study on* Terminalia glaucescens* when they demonstrated that the ethyl acetate extract was more effective than the ethanol extract at the same dose on certain microorganisms. The possible explanation to the difference of activity between the two extracts may be the ability of solvent to solubilize and extract some phytomolecules. Thus, according to Cowan [55], during the liquid-liquid extraction, phytomolecules are distributed between the solvents according to their polarity and solubility. It can be thus concluded that the active antimicrobial compounds contained in the bark of* C. nitida* are more soluble in the ethyl acetate solvent than ethanol one. The active antistaphylococcal principles contained in the bark of* C. nitida* are more concentrate by ethyl acetate.

Analyzing Figure 2, it appears that the inhibition zone diameters were not significantly different independently of the extract solvent regardless of duration (24 h and 48 h) on both reference (Figures 2(a) and 2(b)) and food isolated strains (Figures 2(c) and 2(d)). Our results are different from those of Arekemase et al. [56] in their study when they observed a significant difference of inhibition diameters in the time. We can notice that Arekemase et al. [56] used 10 times higher concentration to the one we used; that may thus be one of the reasons. Indeed, with the highest concentration, it is possible to have an increase of the inhibition diameter because the active antimicrobial substance is in excess.

The minimum inhibitory concentration (MIC) was variable depending on the strains and extracts (Table 3). Our found concentrations were higher than those reported by Dahake et al. [57] when they proved that* S. aureus* and* Bacillus subtilis* were more sensitive to the ethanol leaf extract of* Anacardium occidentale* with MIC = 15.62 *μ*g/mL. The difference may probably be due to the divergent extraction methods and the different origins of strains used. Indeed, depending on the extraction methods, the antimicrobial agents extracted may have different concentrations. Also, this indicates that* C. nitida*'s bark extracts were less active at lowest concentration compared with those of* A. occidentale*.

The minimum bactericidal concentrations (MBCs) were variable according not only to the strains but also to the type of extracts (Table 4). Our results were not the same as those obtained by Sika et al. [28] reporting a range of 0.078 to 0.625 mg/mL (for reference strains) and from 0.078 to 1.25 mg/mL (for meat isolated* Staphylococcus *strains) as MBCs during an* in vitro* test on* A. occidentale* extracts. As the used strains are from the same origin, the difference may be due to the phytochemical composition of the extracts.

The ratio between the MIC and MBC shows that, according to the strains, extracts have both bactericidal and bacteriostatic effects on reference and food isolated strains (Table 5). The two kinds of activities were already reported in previous studies on some plants such as* Erythrina senegalensis* [58] and* A. occidentale* [28].

The antifungal activity of* Cola nitida*'s bark extracts (Figure 3) was statistically variable in regard of the used fungal strains (*P* = 0.0016). Independently of the extracts,* A. tamarii* was the most resistant strain. Indeed, the limits established by Reyes et al. [59] allow concluding that our extracts have antifungal activity against the three tested strains. In comparison with studies performed using* C*.* nitida*'s seeds on one hand on* Aspergillus niger* and* Aspergillus fumigatus* in Nigeria [60] and on the other hand in Côte d'Ivoire against* Fusarium oxysporum* [61] we can conclude that the bark of* C. nitida* has an interesting antifungal activity. Indeed, in their study in Nigeria (80 mg/mL) and Cote d'Ivoire (3.04 mg/mL), these authors reported extremely high concentration in comparison to the 1.8 mg/mL we used in our study.

The antioxidant activity of different extracts by both methods (DPPH and ABTS) is reported in Table 6. Our results, with the DPPH method, corroborate those reported in Cameroon by Momo et al. [62] on the stems of* Cola nitida* extracts (ethanol and aqueous extract) and in Nigeria on the seed of* C. nitida* [12]. Indeed, Momo et al. [62] found out that the ethanol extract of* C. nitida* stems has a high DPPH reduction percentage whereas the aqueous extract does not have any reduction activity of DPPH radical. This result suggests that the extraction solvent plays an important role in the scavenging of free radical. In their studies, Ayebe et al. [12] and Momo et al. [62] reported the highest IC_50_ values in comparison to ours. The difference of values may be due to the used organs: bark in our study, seed in Nigeria [12], and stem in Cameroon [62]. To end, we can also notice that the reduction of free radical ability varies from a reference molecule to another and from an extract to another; this variation may be due to the concentration of antiradical molecules and assays conditions. Therefore, considering the Antiradical Indexes Activity [41], we can conclude that the* Cola nitida* bark ethanol and ethyl acetate extracts have a strong free radical scavenging activity (AAI > 2). Combining the two methods (DPPH and ABTS), we observe that* Cola nitida*'s bark extracts have an excellent reducing free radical activity.

Referring to the values reported by Mousseux [63], the two extracts of* C. nitida*'s bark we tested in our studies (Figure 5) are not toxic at the tested doses. Our result corroborates those found in Nigeria by Ayebe et al. [12] during their study testing the toxicity of* Cola nitida*'s seeds aqueous extract on the rat.

## 5. Conclusion

Through the obtained results, we can say that* Cola nitida*'s bark contains many secondary metabolites dominated by polyphenol compounds. The presence of those compounds confers to* C. nitida*'s bark, through the ethanol and the ethyl acetate extract, some important biological activities. The tested extract displays more bactericidal effect on reference strains than on meat isolated* Staphylococcus* strains. For most of the investigated biological activity, the ethyl acetate extract is more effective than the ethanol extract. The more purified extracts of* C. nitida*'s bark can be useful both in food conservation and in human medicine.

## Figures and Tables

**Table 1:** Phytochemical composition of *Cola nitida*'s bark powder.

Chemical compound	*Cola nitida*'s bark
Alkaloids	**−**
Tannins	**+**
Saponosides (MI)	**+ (167)**
Anthocyanins	**+**
Flavonoids	**+**
Steroids	**−**
Triterpenes	**−**
Coumarin	**−**
Reducing compound	**−**
Glycosides	**+**
Cyanogenic derivate	**−**

+ = presence; − = absence; MI: Moss Index.

**Table 2:** Results of agar disc diffusion assays showing the antibacterial activity of some tested microorganisms in the presence of extracts.

Reference strains	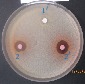	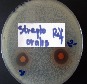	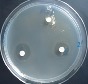	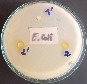	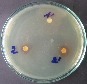
	*Staphylococcus aureus *	*Streptococcus oralis *	*Staphylococcus epidermidis *	*Escherichia coli *	*Pseudomonas aeruginosa *

Meat isolated *Staphylococcus *	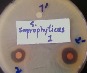	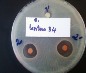	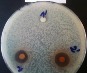	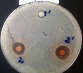	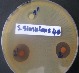
	*Staphylococcus saprophyticus *	*Staphylococcus lentus *	*Staphylococcus haemolyticus *	*Staphylococcus equorum *	*Staphylococcus simulans *

1′: control, **2**: ethanol extracts, and 2′: ethyl acetate extracts.

**Table 3:** Minimum inhibitory concentrations of *C.  nitida*'s bark extract on reference strains and meat isolated *Staphylococcus* strains.

Strains	Minimum inhibitory concentrations (mg/ml)	
	Ethanol extract	Ethyl acetate extract
Reference strains		
*Staphylococcus aureus *	0.312	1.25
*Pseudomonas aeruginosa *	0.625	1.25
*Proteus mirabilis *	1.25	1.25
*Micrococcus luteus *	05	2.5
*Staphylococcus epidermidis *	1.25	0.625
*Proteus vulgaris *	1.25	1.25
*Streptococcus oralis *	0.312	0.625
*Enterococcus faecalis *	0.312	1.25
*Escherichia coli *	—	—
*Candida albicans *	0.625	0.312
Meat isolated *Staphylococcus* strains		
*S. sciuri *	0.625	0.312
*S. aureus *	1.25	0.312
*S. simulans *	0.312	0.152
*S. cohnii *	0.625	0.312
*S. xylosus *	1.25	0.312
*S. equorum *	0.156	0.078
*S. saprophyticus *	0.312	0.078
*S. haemolyticus *	0.078	0.625
*S. lentus *	0.156	0.078

**Table 4:** Minimum bactericidal concentrations of *C.  nitida*'s bark extract on reference strains and meat isolated *Staphylococcus* strains.

Strains	Minimum bactericidal concentrations (mg/ml)	
	Ethanol extract	Ethyl acetate extract
Reference strains		
*Staphylococcus aureus *	2.5	1.25
*Pseudomonas aeruginosa *	2.5	2.5
*Proteus mirabilis *	10	05
*Micrococcus luteus *	*>*20	>20
*Staphylococcus epidermidis *	2.5	2.5
*Proteus vulgaris *	10	05
*Streptococcus oralis *	05	2.5
*Enterococcus faecalis *	2.5	1.25
*Escherichia coli *	—	—
*Candida albicans *	2.5	2.5
Meat isolated *Staphylococcus* strains		
*S. sciuri *	05	0.625
*S. aureus *	2.5	2.5
*S. simulans *	2.5	2.5
*S. cohnii *	05	2.5
*S. xylosus *	05	05
*S. equorum *	0.625	0.625
*S. saprophyticus *	2.5	0.625
*S. haemolyticus *	2.5	2.5
*S. lentus *	2.5	0.625

**Table 5:** Bactericidal and bacteriostatic effects of *Cola nitida* extracts on reference strains and meat isolated strains.

Strains	CMB/CMI	
	Ethanol extract	Ethyl acetate extract
Reference strains		
*Staphylococcus aureus *	8.01	1^*^
*Pseudomonas aeruginosa *	4	2^*^
*Proteus mirabilis *	8	4
*Micrococcus luteus *	*>*4	>8
*Staphylococcus epidermidis *	2^*^	2^*^
*Proteus vulgaris *	8	4
*Streptococcus oralis *	16.02	4
*Enterococcus faecalis *	8.01	1^*^
*Escherichia coli *	—	—
*Candida albicans *	4	8.01
Meat isolated *Staphylococcus* strains		
*S. sciuri *	8	2^*^
*S. aureus *	2^*^	8.01
*S. simulans *	8.01	16.44
*S. cohnii *	4	16.02
*S. xylosus *	8	8.01
*S. equorum *	4.01	8.01
*S. saprophyticus *	8.01	8.01
*S. haemolyticus *	32.05	4
*S. lentus *	16.02	8.01

With ∗ = bactericidal effects; without ∗ = bacteriostatic effects.

**Table 6:** Parameters of free radical scavenging activity by DPPH radical and ABTS methods.

	DPPH		ABTS
	IC_50_	AAI	*C*
	(*μ*g·*μ*l^−1^)		(*μ*molEqAA·g^−1^)
Ethanol extract	9.00 ± 1.73	5.71 ± 1.23	49.72 ± 0.35
Ethyl acetate extract	4.53 ± 0.98	11.02 ± 1.49	53.39 ± 0.0
Quercetin	4.51 ± 0.35	11.11 ± 0.85	—
Gallic acid	0.73 ± 0.12	62.74 ± 5.54	—
Ascorbic acid	—	—	35.02 ± 0.73

## References

[1] Sofowora A. (1993). *Medicinal Plants and Traditional Medicine in Africa*.

[2] Iniaghe O. M., Malomo S. O., Adebayo J. O. (2009). Proximate composition and phytochemical constituents of leaves of some *Acalypha* species. *Pakistan Journal of Nutrition*.

[3] Diallo A. M. (2005). *Etude des plantes médicinales de niafunke (région Tombouctou) Phytochimie et pharmacologie de MaeruacrassifoliaForsk (Capparidacée) [Thèse de Doctorat]*.

[4] WHO (2002). *Strategie de l’OMS pour la medicine traditionnelle pour 2002–2005*.

[5] Akoegninou A., van der Burg W. J., van der Maesen L. J. G. (2006). *Flore Analytique du Bénin*.

[6] Kouame K., Scande M. (2006). *Cola nitida* (vent) Schott & Endl. *Seed Leaflet, Forest & Landscape*.

[7] Mokwunye F. C. (2009). *Functional characterisation of kola nut powder for beverage production [M.S. thesis]*.

[8] Nzekwu O. (1961). Kola nut. *Nigeria Magazine*.

[9] Jayeola C. O. (2001). Preliminary studies on the use of kolanuts (*Cola nitida*) for soft drink production. *Journal of Food Technology in Africa*.

[10] Javies G. The rise and fall of cocaine cola. http://www.unz.org/Pub/LewRockwell2002may-00039.

[11] Lowor S. T., Aculey P. C., Assuah M. K. (2010). Analysis of some quality indicators in cured *Cola nitida* (Vent). *Agriculture and Biology Journal of North America*.

[12] Ayebe E. K., Yapi H. F., Edjeme A. A. (2012). *In vivo*, *in vitro* antioxidant activity assessment & acute toxicity of aqueous extract of *Cola nitida* (Sterculiaceae). *Asian Journal of Biochemical and Pharmaceutical Research*.

[13] Muhammad S., Fatima A. (2014). Studies on phytochemical evaluation and antibacterial properties of two varieties of kolanut (*Cola nitida*) in Nigeria. *Journal of Biosciences and Medicines*.

[14] Ryan K. J., Ray C. G. (2004). *Sherris Medical Microbiology*.

[15] Vincenot F., Saleh M., Prévost G. (2008). Les facteurs de virulence de *Staphylococcus aureus*. *Revue Francophone des Laboratoires*.

[16] Chang S., Sievert D. M., Hageman J. C. (2003). Infection with vancomycin-resistant *Staphylococcus aureus* containing the *vanA* resistance gene. *The New England Journal of Medicine*.

[17] Malíková L., Sedláček I., Novákovâ D., Němec M. (2007). Ribotyping and biotyping of *Staphylococcus epidermidis* isolated from hospital environment. *Folia Microbiologica*.

[18] Zhang K., Sparling J., Chow B. L. (2004). New quadriplex PCR assay for detection of methicillin and mupirocin resistance and simultaneous discrimination of *Staphylococcus aureus* from coagulase-negative staphylococci. *Journal of Clinical Microbiology*.

[19] Morellion P., Que Y. A., Glauser M. P., Mandel G. L., Bennett J. E., Dolin R. (2005). Staphylococcus aureus. *Principles and Practice of Infectious Diseases*.

[20] Akcam F. Z., Tinaz G. B., Kaya O., Tigli A., Ture E., Hosoglu S. (2009). Evaluation of methicillin resistance by cefoxitin disk diffusion and PBP2a latex agglutination test in *mecA*-positive *Staphylococcus aureus*, and comparison of *mecA* with *femA*, *femB*, *femX* positivities. *Microbiological Research*.

[21] Zohra M. (2013). *Etude Phytochimique et Activités Biologiques de quelques Plantes médicinales de la Région Nord et Sud-Ouest de l’Algérie [Thèse de doctorat]*.

[22] Bagchi D., Sen C. K., Bagchi M., Atalay M. (2004). Anti-angiogenic, antioxidant, and anti-carcinogenic properties of a novel anthocyanin-rich berry extract formula. *Biochemistry*.

[23] Patthamakanokporn O., Puwastien P., Nitithamyong A., Sirichakwal P. P. (2008). Changes of antioxidant activity and total phenolic compounds during storage of selected fruits. *Journal of Food Composition and Analysis*.

[24] Sokpon N., Ouinsavi C. (2002). Utilisation de *Khaya senegalensis* en médecine traditionnelle au Bénin. *Revue de Médecine et Pharmacopée Africaines*.

[25] Biecke B. Ethnobotanishestudie van geneeskrachtigeplnten in Manigri en Igbére, Bénin.

[26] Adjanohoun E., Adjakidjè V., Ahyi M. R. A. (1989). *Contribution aux Etudes Ethnobotaniques et Floristiques en République Populaire du Bénin*.

[27] Sessou P., Farougou S., Azokpota P. (2012). *In-vitro* antifungal activity of essential oil of *Pimenta racemosa* against fungal isolates from wagashi, a traditional cheese produced in Benin. *International Journal of Natural and Applied Sciences*.

[28] Sika C. K., Sina H., Adoukonou-Sagbadja H. (2014). Antimicrobial activity of *Anacardium occidentale* L. leaves and barks extracts on pathogenic bacteria. *African Journal of Microbiology Research*.

[29] Houghton P. J., Raman A. (1998). *Laboratory Hand Book for the Fractionation of Natural Extracts*.

[30] Sanogo R., Diallo D., Diarra S., Ekoumou C., Bougoudogo D. (2006). Activité antibactérienne et antalgique de deux recettes traditionnelles utilisées dans le traitement des infections urinaires et la cystite au Mali. *Mali Médical*.

[31] N'Guessan J. D., Bidié A. P., Lenta B. N., Weniger B., André P., Guédé-Guina F. (2007). *In vitro* assays for bioactivity-guided isolation of anti salmonella and antioxidant compounds in *Thonningia sanguinea* flowers. *African Journal of Biotechnology*.

[32] Attien P., Sina H., Moussaoui W. (2013). Prevalence and antibiotic resistance of *Staphylococcus* strains isolated from meat products sold in Abidjan streets (Ivory Coast). *African Journal of Microbiology Research*.

[33] Bauer A. W., Kirby W. M., Sherris J. C., Turck M. (1966). Antibiotic susceptibility testing by a standardized single disk method. *The American Journal of Clinical Pathology*.

[34] Gupta S., Ali M., Alam M. S. (1993). A naphthoquinone from *Lawsonia inermis* stem bark. *Phytochemistry*.

[35] Saha A., Rahman M. S. (2008). Antimicrobial activity of crude extract from *Calycopteris floribunsa*. *Bangladesh Journal of Microbiology*.

[36] Farshori N. N., Al-Oqail M. M., Al-Sheddi E. S., Siddiqui M. A., Rauf A. (2013). Antimicrobial potentiality of *Polyalthia longifolia* seed oil against multi drug resistant (MDR) strains of bacteria and fungus of clinical origin. *African Journal of Microbiology Research*.

[37] Kawsar S. M. A., Huq E., Nahar N. (2008). Cytotoxicity assessment of the aerial parts of *Macrotyloma uniflorum* linn. *International Journal of Pharmacology*.

[38] Kumar S., Thomas A., Sahgal A., Verma A., Samuel T., Pillai M. K. K. (2002). Effect of the synergist, piperonyl butoxide, on the development of deltamethrin resistance in yellow fever mosquito, *Aedes aegypti* L. (Diptera: Culicidae). *Archives of Insect Biochemistry and Physiology*.

[39] Dohou N., Yamni K., Badoc A., Douira A. (2004). Activité antifongique d'extraits de *Thymelaea lythroides* sur trois champignons pathogènes du riz. *Bulletin de la Société de pharmacie de Bordeaux*.

[40] Re R., Pellegrini N., Proteggente A., Pannala A., Yang M., Rice-Evans C. (1999). Antioxidant activity applying an improved ABTS radical cation decolorization assay. *Free Radical Biology & Medicine*.

[41] Guenne S., Ouattara N., Hilou A., Millogo J. F., Nacoulma O. G. (2011). Antioxidant, enzyme inhibition activities and polyphenol contents of three *Asteraceae* species used in Burkina Faso traditionally medicine. *International Journal of Pharmacy and Pharmaceutical Sciences*.

[42] Scherer R., Godoy H. T. (2009). Antioxidant activity index (AAI) by the 2,2-diphenyl-1-picrylhydrazyl method. *Food Chemistry*.

[43] Schmeda-Hirschmann G., Rodriguez J. A., Theoduloz C., Astudillo S. L., Feresin G. E., Tapia A. (2003). Free-radical scavengers and antioxidants from *Peumus boldus* Mol. (‘Boldo’). *Free Radical Research*.

[44] Bruneton J. (1999). *Les Tanins*.

[45] Ortuño A., Báidez A., Gómez P. (2006). *Citrus paradisi* and *Citrus sinensis* flavonoids: their influence in the defence mechanism against *Penicillium digitatum*. *Food Chemistry*.

[46] N’Guessan K., Kadja B., Zirihi G. N., Traoré D., Aké-Assi L. (2009). Screening phytochimique de quelques plantes médicinales ivoiriennes utilisées en pays Krobou (Agboville, Côte-d’Ivoire). *Sciences & Nature*.

[47] Sonibare M. A., Soladoye M. O., Esan O. O., Sonibare O. O. (2009). Phytochemical and antimicrobial studies of four species of Cola Schott & Endl. (Sterculiaceae). *African Journal of Traditional, Complementary and Alternative Medicines*.

[48] Cybulski W. J., Peterjohn W. T., Sullivan J. H. (2000). The influence of elevated ultraviolet-B radiation (UV-B) on tissue quality and decomposition of loblolly pine (*Pinus taeda* L.) needles. *Environmental and Experimental Botany*.

[49] Braga M. R., Aidar M. P. M., Marabesi M. A., de Godoy J. R. L. (2006). Effects of elevated CO_2_ on the phytoalexin production of two soybean cultivars differing in the resistance to stem canker disease. *Environmental and Experimental Botany*.

[50] Tsukaya H., Tsujino R., Ikeuchi M. (2007). Morphological variation in leaf shape in *Ainsliaea apiculata* with special reference to the endemic characters of populations on Yakushima Island, Japan. *Journal of Plant Research*.

[51] Folkers A., Hüve K., Ammann C. (2008). Methanol emissions from deciduous tree species: dependence on temperature and light intensity. *Plant Biology*.

[52] Shen H., Tang Y., Muraoka H., Washitani I. (2008). Characteristics of leaf photosynthesis and simulated individual carbon budget in *Primula nutans* under contrasting light and temperature conditions. *Journal of Plant Research*.

[53] Indabawa I. I., Arzai A. H. (2011). Antibacterial activity of *Garcinia kola* and *Cola nitida* seed extracts. *Bayero Journal of Pure and Applied Sciences*.

[54] Bolou G. E. K., Attioua B., N'Guessan A. C., Coulibaly A., N'Guessan J. D., Djaman A. J. (2011). Évaluation *in vitro* de l'activité antibactérienne des extraits de *Terminalia glaucescens* planch. sur *Salmonella typhi* et *Salmonella typhimurium*. *Bulletin de la Société Royale des Sciences de Liège*.

[55] Cowan M. M. (1999). Plant products as antimicrobial agents. *Clinical Microbiology Reviews*.

[56] Arekemase M. O., Oyeyiola G. P., Aliyu M. B. (2011). Antibacterial activity of *Anacardium occidentale* on some enterotoxin producing bacteria. *Internationnal Journal of Biology*.

[57] Dahake A. P., Joshi V. D., Joshi A. B. (2009). Antimicrobial screening of different extract of *Anacardium occidentale* Linn. leaves. *International Journal of ChemTech Research*.

[58] Dramane S., Mamidou K. W., Kagoyire K. (2010). Evaluation des activites antimicrobiennes et anti-radicaux libres de quelques taxons bioactifs de Côte. *European Journal of Scientific Research*.

[59] Reyes C. R., Quiroz V. R. I., Jiménez E. M., Navarro-Ocaňa A., Cassani H. J. (1997). Antifungal activity of selected plant secondary metabolites against *Coriolus versicolor*. *Journal of Tropical Forest Products*.

[60] Kanoma A. I., Muhammad I., Ibrahim I. D., Shehu K., Maishanu H. M., Isah A. D. (2014). Phytochemical screening of various species of cola nut extracts for antifungal activity against phytopathogenic fungi. *The American Journal of Biology Life Sciences*.

[61] Koné D., Brahima C., Odjochounou B. J., Odile Carisse (2010). Fungicides and biological products activities towards fungi causing diseases on banana and vegetable in Cote d'Ivoire. *Fungicides*.

[62] Momo C. E. N., Ngwa A. F., Dongmo G. I. F., Oben J. E. (2009). Antioxidant properties and *α*-amylase inhibition of *Terminalia superba*, *Albizia* sp., *Cola nitida*, *Cola odorata* and *Harungana madagascarensis* used in the management of diabetes in Cameroon. *Journal of Health Science*.

[63] Mousseux M. (1995). *Test de toxicité sur larves de Artemia salina entretien d’un élevage de balanes*.

